# Epidemiology of Hepatitis E Virus in an Urban Community in Dhaka City

**DOI:** 10.5005/jp-journals-10018-1087

**Published:** 2014-01-22

**Authors:** Salimur Rahman, Mamun-Al-Mahtab, Munira Jahan, Shahina Tabassum, Sheikh Mohammad Fazle Akbar

**Affiliations:** 1 Department of Hepatology, Bangabandhu Sheikh Mujib Medical University, Dhaka, Bangladesh; 2 Department of Hepatology, Bangabandhu Sheikh Mujib Medical University, Dhaka, Bangladesh; 3 Department of Virology, Bangabandhu Sheikh Mujib Medical University, Dhaka, Bangladesh; 4 Department of Virology, Bangabandhu Sheikh Mujib Medical University, Dhaka, Bangladesh; 5 Department of Medical Sciences, Toshiba General Hospital, Tokyo, Japan

**Keywords:** Hepatitis E, Prevalence, Urban population.

## Abstract

**Introduction:**

Hepatitis E virus (HEV) is endemic in Bangladesh and sporadic and epidemic outbreaks of acute hepatitis E occur in this country almost regularly. Although the real magnitude of HEV prevalence has not been documented in Bangladesh, HEV infections and HEV-related acute hepatitis of Bangladeshi origin have been reported from different parts of the world.

**Methods:**

The study was conducted in Mirpur area of Dhaka city, which is a major residential area of the capital of Bangladesh. Three hundred adults were randomly included in the study. None had any history of jaundice or complains of liver diseases.

**Results:**

The study revealed 30% prevalence of HEV in this population. The prevalence increased with age, but there was no gender difference.

**Conclusion:**

HEV is a highly prevalent disease in Bangladesh as elsewhere in the developing world. Since there is no specific treatment for HEV, improvement of personal hygiene and ensuring supply of safe food and drinking water remain most important approach to sustain the virus.

**How to cite this article:** Rahman S, Mahtab MA, Jahan M, Tabassum S, Akbar SMF. Epidemiology of Hepatitis E Virus in an Urban Community in Dhaka City. Euroasian J Hepato-Gastroenterol 2014; 4(1):4-6.

## INTRODUCTION

Hepatitis E virus (HEV) is a spherical, nonenveloped, single-stranded RNA virus^1^ having 5 genotypes designated from I-V. Of these, genotype I has been reported from Asia and Africa, genotype II from United States, genotype III from Mexico, genotype IV from China and genotype V from Europe.^2-4^

HEV is excreted in feces and transmission is predominantly by fecal-oral route, usually through contaminated water.^5^ Most HEV outbreaks have been reported during rainy seasons and after floods.^6^ There is also evidence of blood-borne transmission of HEV and zoonotic transmission of HEV in advanced countries.^7-9^ In between outbreaks, subclinical HEV-infections in humans and/or animals act as reservoir for the virus.^10^

Hepatitis E virus is endemic in Bangladesh and sporadic and epidemic outbreaks of acute hepatitis E occur in this country almost regularly. Some hospital-based studies have also shown that HEV is main etiological agent of acute hepatitis,^11^ fulminant hepatitis,^12,13^ and acute on chronic liver failure^14^ at Bangladesh. However, little has been explored about prevalence of HEV among general population. One study by Labrique et al^15^ exposed that about 23% of apparently healthy population of rural Bangladesh were seroposi-tive for HEV. However, there is a paucity of information about sero-prevalence of HEV among urban population of Bangladesh. This is an important issue because the route of transmission of HEV may be different between rural and urban populations. In addition, most of the epidemics of HEV have been reported from urban areas. The study was accomplished at Dhaka, the capital of Bangladesh.

## METHODS

The study was conducted in Mirpur area of Dhaka city, which is a major residential area of the capital of Bangladesh. Ethical approval of the study was obtained from the Ethical Committee at Viral Hepatitis Foundation of Bangladesh. In this pilot study, a total of300 adults, aged between 18 and 67 years, were enrolled (Table 1). One participant was randomly selected from every one in ten households of the area. The first 300 adults with an equal distribution of male and female were included. Informed written consent was obtained from each participant. Five milliliter of venous blood was obtained from each person with full aseptic precautions. Sera were separated immediately to avoid hemolysis, appropriately labeled and stored at -20°C until assessment. Stored sera were subsequently tested for anti-HEV immunoglobulin G (IgG) by enzyme-linked immunosorbent assay (ELISA) (Institute of Immunology Co. Ltd., Tokyo, Japan).

**Table 1:** **Table 1:** Characteristics of study population (n = 300)

*Age (years)*		*No.*		*Male*		*Female*		*Anti-HEV IgG positive*		*Percentage (%)*	
18-30		87		39 (42.8%)		48 (57.2%)		24 (27.6%) (M:F = 9:15)		M:F = 37.5:62.5	
31-40		60		27 (45%)		33 (55%)		24 (40%) (M:F = 9:15)		M:F = 37.5:62.5	
41-50		84		48 (57.1%)		36 (42.9%)		18 (21.4%) (M:F = 15:3)		M:F = 83.3:16.7	
51-60		42		24 (57.1%)		18 (42.9%)		15 (35.7%) (M:F = 12:3)		M:F = 80:20	
>60		27		12 (44.4%)		15 (55.6%)		09 (52.9%) (M:F = 3:6)		M:F = 33.3:66.7	

HEV: Hepatitis E virus, IgG: Immunoglobulin

## RESULTS

The age of subjects ranged from 18 to 67 years. Of them, 150 were males and rest 150 females. None had any history of jaundice or complains of liver diseases. Also, there was no subjective symptom of liver diseases in any patient. Ninety (30%) were tested positive for anti-HEV IgG. The prevalence was lowest among 18 to 30 years age group where it was 27.6%, rising more or less steadily with age peaking at 52.9% among those above 60 years of age. The only exception is the age group 41 to 50 years, where the prevalence declines to 21.4%.

Both sexes are affected equally and no sex predominance was noted. The figures vary between men and women in different age groups. For example, the male prevalence is almost 4 times higher than females in those between 41 and 60 years of age, but in case of females over 60 years, the prevalence is almost 3 times higher than males. And the prevalence of anti-HEV IgG is roughly double in females than that in males among the 18 to 40 years age group.

## DISCUSSION

Although HEV has been shown to be the main etiological agent for acute hepatitis,^11^ fulminant hepatitis^12,13^ and acute on chronic liver failure,^14^ prevalence of HEV has seldom been studied in Bangladesh. This study is the second that assessed the prevalence of HEV among apparently healthy individuals. Labrique et al^15^ have shown that about 22% of rural Bangladeshi was expressing IgG type antibody to HEV. We have shown that 27% of urban Bangladeshi showed evidence of past HEV infection. Taken together, it can be postulated that HEV is highly endemic in both rural and urban population of Bangladesh, although the source of water and means of disposal of sewerage show marked differences among rural and urban areas of Bangladesh.

Although the real magnitude of HEV prevalence has not been documented by epidemiological studies, HEV infections and HEV-related acute hepatitis of Bangladeshi origin have been reported from different parts of the world. The first report of HEV-related acute hepatitis E has been among the inhabitants. The Netherlands who traveled to Bangladesh in early 1990s.^16^ Subsequently, acute hepatitis E has been reported from nationals of Spain, Japan and Italy^17-20^ who traveled to Bangladesh. In addition, acute hepatitis E has also been recorded among United Nation Peace keepers of Bangladeshi origin at Haiti.^21,22^

The present study and that of Labrique et al^15^ have provided some clue regarding HEV infection among people traveling to Bangladesh. Usually, travelers try to take care of different public health measures during their travel. However, as one of every 3 to 4 apparently healthy Bangladeshi is infected with HEV, acquisition of HEV is a common factor for travelers coming to Bangladesh.
